# Hsp90 Inhibitor; NVP-AUY922 in Combination with Doxorubicin Induces Apoptosis and Downregulates VEGF in MCF-7 Breast Cancer Cell Line

**DOI:** 10.31557/APJCP.2020.21.6.1773

**Published:** 2020-06

**Authors:** Mahshid Mohammadian, Sadegh Feizollahzadeh, Reza Mahmoudi, Attabak Toofani Milani, Soheila Rezapour Firouzi, Bahareh Karimi Douna

**Affiliations:** 1 *Department of Medical Laboratory Sciences, School of Allied Medical Sciences, Urmia University of Medical Sciences, ,Urmia, Iran. *; 2 *Department of Toxicology, Faculty of Pharmacy, Islamic Azad University, Shahreza, Iran. *; 3 *Department of Clinical Biochemistry, School of Medicine, Urmia University of Medical Sciences, Urmia, Iran. *; 4 *Cellular and Molecular Research Center, Cellular and Molecular Medicine Institute, Urmia University of Medical Sciences, Urmia, Iran. *; 5 *Department of Life Science Engineering, Faculty of New Sciences and Technologies, University of Tehran, Tehran, Iran. *

**Keywords:** Breast cancer, doxorubicin, NVP-AUY922, Caspase 3, vascular endothelial growth factor, apoptosis

## Abstract

**Objective:**

Breast cancer is one of the most prevalent malignancies and leading causes of females’ mortality worldwide. Because of resistance to various treatment options, new treatments based on molecular targeting has introduced as noticeable strategies in cancer treatment. In this regard, heat shock protein 90 (Hsp90) inhibitors are proposed as effective anticancer drugs. The goal of the study was to utilize a combination of the doxorubicin (DOX) and NVP-AUY 922 on the MCF-7 breast cancer model to investigate the possible cytotoxic mechanisms.

**Methods:**

MCF-7 breast cancer cell line was prepared and treated with various concentrations of DOX and NVP-AUY922 in single-drug treatments. We investigated the growth-inhibitory pattern by MTT assay after continuous exposure to NVP-AUY922 and DOX in order to determine dose-response. Then the combinatorial effects were evaluated in concentrations of 0.5 × IC_50_, 0.2 × IC_50_, 1 × IC_50_ and, 2 × IC_50_ of each drugs. Based on MTT results of double combinations, low effective doses were selected for Real-time PCR [caspase3 and vascular endothelial growth factor (VEGF)] and caspase 3 enzyme activity.

**Results:**

A dose-dependent inhibitory effects were presented with increasing the doses of both drugs in single treatments. The upregulation of caspase 3 and downregulation of VEGF mRNA were observed in double combinations of NVP-AUY922 and DOX versus single treatments. Also, in these combinations in low doses of examined drugs (0.5 × IC_50_, 0.2 × IC_50_), higher caspase 3 activity were presented in comparison to single treatments (p<0.05).

**Conclusions:**

Our findings indicate an effective action of NVP-AUY922 in combined with DOX in this cell line. These results can predict the treatment outcome in this model.

## Introduction

Breast cancer is considered as a one of the most prevalent malignancies and leading causes of females’ mortality worldwide (Maroufi et al., 2020). The resistance to different treatments, including chemotherapy, is not commonly exclusive. It appears that the tumor could be resistant to multiple treatment options. Nevertheless, the underlying mechanisms are complicated and need more studies. Moreover, drug resistance is a common problem in treatments of all breast cancer types, although the various treatment strategies could be applied (Chun et al., 2017). In this regard, targeted therapy in various types of malignancies including solid tumors considered as promising treatment options (Jensen et al., 2008). Indeed, cancer cells often use the specified molecular mechanism to increase their capacity for growth ( Arshad et al., 2018). One main target in effective cancer therapies is to induction of effective cytotoxic response after chemotherapy using drug response assays (Mohamadi et al., 2017; Mohammadian et al., 2019; Mohammadian et al., 2017; Moradi et al., 2019; Zeynali et al., 2018). Doxorubicin (DOX) is one of the furthermost effective chemotherapeutic agents in the treatment of breast cancer. Unfortunately, resistance to DOX is mutual and considered as a significant problem to successful treatment. In this regard, evaluating the new approaches which are related to treatment response may permit effective therapy to be proposed (Smith et al., 2006).

The heat shock protein 90 (Hsp90) as a main molecular chaperone has an essential role in functionality of its client proteins in cells. The Hsp90 client proteins comprise the numerous signal transducing molecules including protein kinases (Miyata et al., 2013). The Hsp90 machinery plays an important roles in the folding, maturation, and assembly of several proteins that involved in cell cycle control. Indeed, HSP90 as multichaperone complex has a significant function in cancer growth and could be considered as a promising target for effective cancer treatment. (Mayor-López et al., 2014; Miyata et al., 2013). So, Hsp90 inhibition with specific inhibitors including geldanamycin and its derivatives decreases the tumor growth. Hsp90 inhibitors can be potential cancer treatment options (Miyata et al., 2013). 

the novel small molecule NVP-AUY922 as highly potent HSP90 inhibitor, has acceptable pharmacological and pharmaceutical properties, and show potent antitumor activity in human cancer cell models with inhibition of cell proliferation in low dose potency (Jensen et al., 2008).

Apoptosis is programmed cell death that has a main role in the assessment of response to the cytotoxic and tumor cell damage. Caspases are main effectors of apoptosis proces that among these mediators, caspase-3 is the central protease activator with critical role in apoptosis induction (Moradi et al., 2019). On the other hands, angiogenesis is essential to tumor growth and invasion. The vascular endothelial growth factor (VEGF) signaling pathway has essential role in controlling the tumor angiogenesis. VEGF as a therapeutic target has been confirmed in different types of human malignancies (Niu et al., 2010).

So, in the present study, we examined the antitumor effects of NVP-AUY922 and DOX in combination cases with targeting angiogenesis and apoptosis main mediators in MCF-7 breast cancer cells.

## Materials and Methods

*Cell culture, drug treatments and MTT assay*

The MCF-7 cell line was obtained from the Pasteur Institute (Tehran, Iran). 

Cells were seeded in Dulbecco’s Modified Eagle Medium containing 10% fetal bovine serum and 1% of penicillin and streptomycin, with 95% humidity and 5% CO2 at 37°C. 

DOX and NVP-AUY922 were provided by Sigma and LC companies respectively. DOX and NVP-AUY922 stock solution was prepared and kept at 4 and -20 ° C, respectively. 

DOX were applied into the culture medium at the concentrations of 0.1, 0.2, 0.25, 0.5, 1, 2, 3 and 5 µM. Also, MCF-7 cells treated with NVP-AUY922 in the doses of 1, 2, 4, 5, 8, 10, 20, 40 and 50 nM,. 

Cytotoxic effects of DOX and NVP-AUY922 on MCF-7 cells were assessed using the MTT test as describe in manufacture protocol (Kia zist, Iran). Briefly, 1×10^4 ^viable cells were cultured in 96-well culture plates with and without examined drugs in a final volume of 100 μl, as triplicates. After 24 h, drugs were added. Cells were allowed to grow in the following 24 h after treatments. The cell viability test performed, and the dose-response curve were drown. The IC_50_ concentrations were assessed by Compusyn software. In following, the various doses (2× IC_50_, 1× IC_50_, IC_50_, 0.5× IC_50_, and 0. 2× IC_50_) of each drugs were examined in double combinations, and effective dosages for Real-Time polymerase chain reaction (PCR) and caspase3 activity were selected(0.5 × IC_50_ and 0.2 × IC_50 _of each drugs in combinatorial cases). Definitely, to evaluate the combined effects of these drugs, we utilized lower effective treatments of them that approved with MTT assay (0.5× IC_50_ and 0. 2× IC_50_) for further studies.

*RNA extraction and Real-Time PCR*

In this study, 24 h After various treatments, total RNA was extracted by using the RNA extraction kit (Gene all,South Korea) according to the manufacturer’s protocol. Reverse transcription was carried out to synthesize the cDNA based on the manufacturer’s instructions. Then cDNA was subjected to Real-Time PCR assay. Indeed, the expression of VEGF and caspase 3 at the mRNA levels was studied. The specific primers sequences of caspase-3 (Atari-Hajipirloo et al., 2017), VEGF (Zeynali et al., 2018) and β-actin ( Atari-Hajipirloo et al., 2017) as a housekeeping gene presented in Table 1. 

Real-time PCR was carried out by Real Q Plus 2x Master Mix Green (Amplicon, Denmark). The PCR conditions were as follow: 15 min at 95°C, then 40 cycles of 95°C for 20 sec; 58°C for 60 sec and 72°C for 5 minutes for VEGF (7)(Zeynali et al., 2018) and at 30 cycles of denaturation for 30 s at 95°C, annealing for 30 seconds at 59°C, and extension for 30 seconds at 72°C for caspase3. Levels of caspase 3 and VEGF were normalized to β-actin (housekeeping gene). The Ct (threshold cycle) value of each sample was measured. Results were calculated using the 2^-ΔΔCt^ method.

*Caspase3 enzyme activity assay*

The caspase-3 colorimetric assay kit (Abnova) was utilized to detect the caspase-3 enzyme activity. Brieﬂy, after 24 h of different treatments, the supernatant was removed, and cells were trypsinized. Then cells were collected and centrifuged at 14,000 rpm for 5 min. in following, cell lysis buffer was added, and cells were retained on ice and centrifuged. Protein concentration was measured with the Bradford method (16)(Bradford, 1976). Proteins (50 μg) diluted by addition of cell lysis buffer. Then 50 μL 2× reaction buffer and 5 μL DEVD-pNA 4 mM substrate was added. The subjected plates were maintained at 37 °C for 2 h and read at 405 nm. The change in caspase 3 activities was assessed by comparing these data with the level of the untreated control.

*Statistical analysis*

Data were assessed with, one-way ANOVA followed by post hoc Tukey test for the study of multiple group comparisons by SPSS statistical software. Results are shown as the mean±Standard deviation. P<0.05 were considered as statistically significant.

## Results

**Figure 1:**
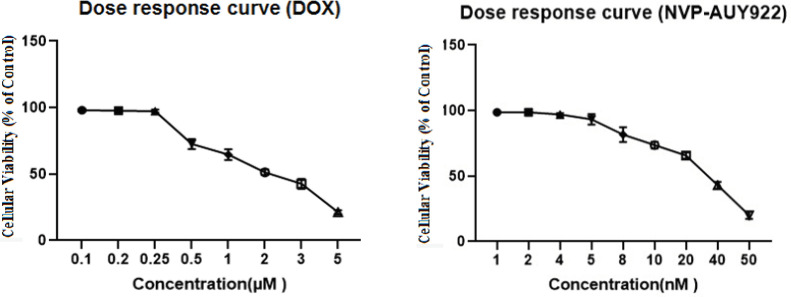
Cellular Viability Results from MTT Analysis (Cytotoxic Effects) of DOX and NVP -AUY922 in Single-Drug Treatments with Different Concentrations after 24 h. Data presented as mean±SD. DOX; Doxorubicin

The effects of the DOX and NVP-AUY922 on cellular viability after 24 h of exposure in the MCF-7 cancer cell line were drawn as the percentage of the viable cells to the untreated control cells. (Figure 1). Our findings show that the exposure of MCF-7 cells to various concentrations of each single drugs decreased cellular viability in the dose dependent manner. Higher IC_50_ values were obtained in DOX compared to NVP-AUY922.

**Figure 2:**
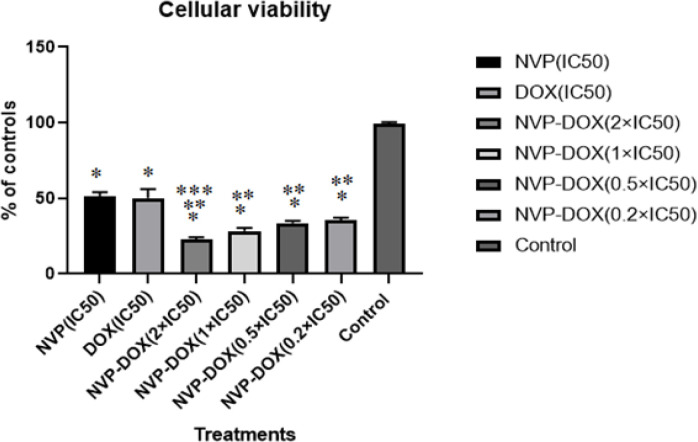
Cellular Viability Assay Results of Various Treatments in Single (IC_50_ Doses) and Double Combinations(2×IC_50_, 1×IC_50_, 0.5×IC_50_ and 0.2×IC_50_) after 24 h. *; significant differences in comparison to untreated control<0.05; **; significant differences in comparison to single treatments (IC50 doses). P<0.05; ***; significant differences in comparison to double treatments(0.5×IC_50_ and 0.2×IC_50_). P<0.05

Various concentrations of DOX and NVP-AUY922 were selected to examine the cytotoxic effects of both drugs in order to assay the effects of drug combinations in MCF-7 cell line. Cellular inhibitory effects of double combinatorial cases were shown in Figure 2.

As presented in Figure 2, in double combination treatments, drug concentrations were 2 × IC_50_, 1× IC_50_, 0.5 × IC_50_, 0.2 × IIC_50_. In all double combinations of DOX and NVP-AUY922, decreased cellular viability were presented compared to single drugs treated cases at IC_50_ doses(P<0.05).

Also, cellular viability decreased significantly in all double combinations compared to untreated controls (P< 0.05). Also, double combinations in 0.5 × IC_50_concentrations had no significant difference with 0.2 × IC_50_ concentration of each drug(P> 0.05). So, in this study, we utilized lower effective doses of both drugs for real-time PCR and caspase 3 enzyme activity (0.5 × IC_50_ and 0.2 × IC_50_). 

**Figure 3:**
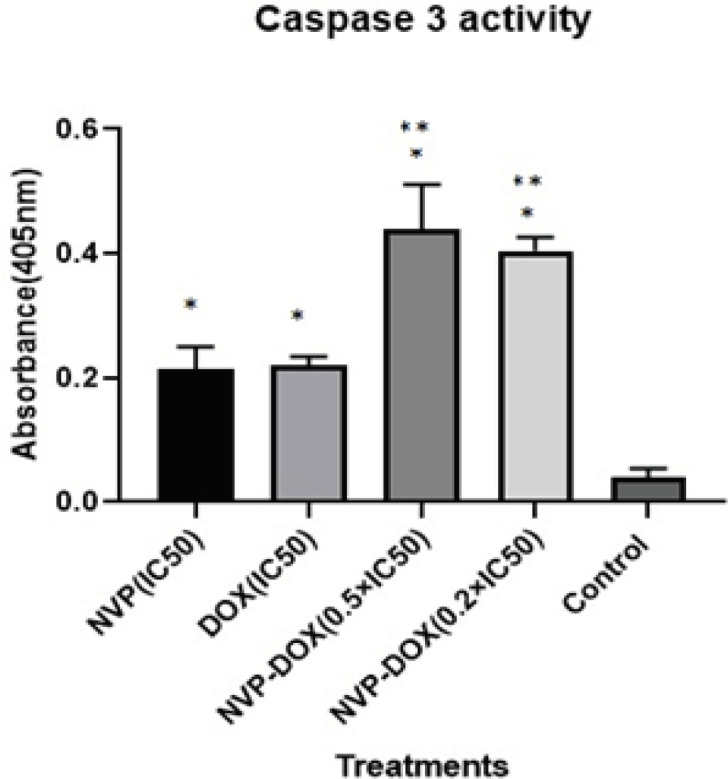
Activities of Caspase 3 in the MCF-7 Breast Cancer Cells after Treatment with the Various Concentrations of NVP-AUY and DOX for 24 h. Each value shows the mean ± SD. *, Indicates a significant difference versus untreated control p<0.05; **, Indicates a significant difference versus single-drugs treatments p<0.05

According to our results (Figure 3), DOX and NVP-AUY922 in single treatments had higher caspase-3 activity in IC_50_ doses versus untreated control cells (p<0.05). In double treatments of both drugs, data showed significant increased caspase 3 enzyme activity compared to control treatment (p<0.05). Likewise, treatment at 0.2×IC_50_ and 0.5×IC_50_ concentrations of each examined drug increased caspase-3 activity significantly (P < 0.05) in compared to single treatments (Figure 3). In comparing these two combinatorial cases (0.2×IC_50_ and 0.5×IC_50_), there were no significant differences (p>0.05).

**Figure 4:**
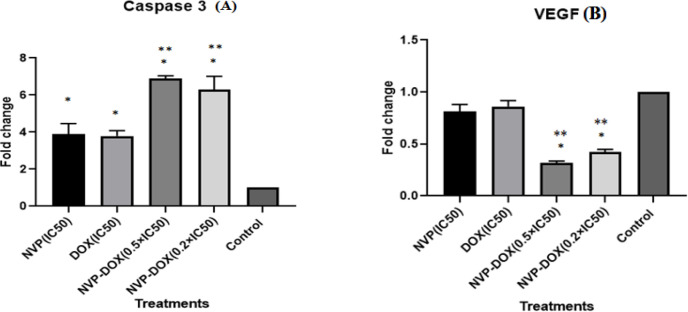
Real Time-PCR Analysis to Determine the Effects of NVP-AUY922 and DOX in Single and Double Drug Treatments on Caspase-3 (A) and VEGF (B) mRNA levels with β-actin as the internal control. Vertical bars show the mean fold changes ± Standard deviation *: significant difference to control.**: significant differences to single-drugs treatment

Based on MTT results, combinations of DOX and NVP-AUY922 at concentrations of 0.2×IC_50_ and 0.5×IC_50_ were used for complementary tests. According to real time PCR results (Figure 4A) there were significant fold increase in caspase-3 mRNA as the result of 0.2×IC_50_ and 0.5×IC_50_ (P< 0.05) treatments of both drugs compared to the control group. Also, the single treatments in IC_50_ doses of single drugs increased the level of caspase-3 mRNA versus the untreated control group (P< 0.05) (Figure 4A). These results showed that NVP-AUY922 elevated caspase-3 gene expression levels more efficiently when combined with DOX. The higher caspase 3-gene expression were observed in double combinations compared to single treatments of each drug (p<0.05). 

Real-time PCR results (Figure 4 B). showed the (insignificant) decreased VEGF gene expression levels in NVP-AUY922 treated cells versus controls (p>0.05). Also, the decreased VEGF levels in a single treatment of DOX at IC_50_ level in compared to untreated cells did not reach a significant level (p>0.05). Double treatments at both examined concentrations showed significantly decreased VEGF gene expression levels compared to controls (p<0.05). In addition, decreased VEGF mRNA were presented in all double combinations versus single treatments (p<0.05) (Figure 4B).

**Table 1:** Primers Sequences to Study the β-actin, VEGF and Caspase-3 Gene Expression Levels in MCF-7 Cell Line

Gene	Primer	Product size (bp)
VEGF (Forward)	5' AGGAGGAGGGCAGAATCATC 3'	141
VEGF (Reverse)	5' GGCACACAGGATGGCTTGAA 3'	
Caspase 3 (Forward)	5'AGAACTGGACTGTGGCATTGAC3'	191
Caspase 3 (Reverse)	5' GCTTGTCGGCATACTGTTTCAG 3'	
β-actin (Forward)	5' CTGGAACGGTGAAGGTGACA 3'	161
β-actin (Reverse)	5' TGGGGTGGCTTTTAGGATGG3'	

## Discussion

Breast cancer is a heterogeneous disease and prevalent malignancy in women worldwide (Amiri et al., 2016; Kanaani, 2017; Maroufi et al., 2020; , Sajjadiyan et al., 2016). Chemotherapy is one of the major treatment strategies for various types of cancers (Kanaani et al., 2017; Poy et al., 2018). But resistance against anticancer drugs considered as a main problems in unsuccessful cancer treatment (Ebrahimifar et al., 2017). 

In this regards, the effectiveness of chemotherapy as an imperative breast cancer treatment strategy has been rigorously restricted because of cancer cells resistance. Since the molecular chaperone HSP90 dysregulated in response to cellular stress (Jokar et al., 2019), the aim of this study was to investigate the anti-cancer effects of HSP90 inhibitor; NVP-AUY922 in combination with conventional standard drug DOX in breast cancer cell line and test their possible mechanism. Human breast cancer cell line was tested for sensitivity against NVP-AUY922 and DOX in vitro. We investigated whether NVP- AUY922, in combination with DOX, could be effectively used to treat breast cancer (in vitro), and also detect the related mechanisms. Based on our results, it seems that NVP-AUY922 might potentiate the effects of DOX. Indeed, combination of NVP-AUY922 and DOX in low doses could inhibit breast cancer cell growth, and upregulate caspase3 as major apoptosis component. Also, these combinations of NVP-AUY922 and DOX in very low concentrations of each drugs down-regulate VEGF as angiogenesis main effector. Indeed these combinations could acts in two way including apoptosis activation and inhibiting angiogenesis which both of these effects confirmed their anti-cancer activities.

VEGF is one of the most potent angiogenic factors that have an essential role in tumor angiogenesis. Increased expression of VEGF was presented in numerous human cancers cells (Adams et al., 2000). Indeed, our double treatments downregulate the VEGF that shows the anti-angiogenic effects of NVP-AUY922 in combined with DOX. Decreases in VEGF may be considered as one of the possible mechanisms of NVP-AUY922- DOX combination in low doses.

Based on the previous report, NVP-AUY922, as a potent small molecule HSP90 inhibitor, display imperative activity against breast cancer. NVP-AUY922 potently binds to HSP90 in a competitive biochemical assay. These effects associated with the NVP-AUY922 ability to interfere with the HSP90-p23 complex in breast cancer cells in culture and xenografts (Jensen et al., 2008).

In other study, HER2-positive breast cancer cell lines showed significant sensitivity to NVP-AUY922 in vitro, with IC_50_ values. NVP-AUY922, in combination with trastuzumab, significantly increased growth inhibition in cell lines, which in accordance with our results (Canonici et al., 2018). Also, in a similar study, NVP-AUY922 treatment decreased VEGF-A excretion in the breast cancer cell line (van Scheltinga et al., 2014).

In a comparable study, Gamitrinib, as HSP90 inhibitor in combination with-doxorubicin, showed increased cytotoxicity in various cancer cell lines, including breast cancer cell lines, which is parallel to our results. Also, drug combination increased caspase activity and cell death in breast cancer cells (Park et al., 2014). Similarly, Ganetespib, as HSP90 inhibitor, potentiated the DOX cytotoxic effects by increased mitotic arrest and DNA damage (Proia et al., 2014). Based on author knowledge, we didn’t find any similar study to compare with our results. 

Our data showed the anti-cancer effects of NVP-AUY922 in combinatorial cases with DOX. The effectiveness of NVP-AUY922 in combination with DOX should be evaluated in clinical practice. To our knowledge these combinations has not yet been investigated in humans and assessment of the other related mechanism should be conducted by preclinical studies. 

In conclusion, our results indicate an effective action of NVP-AUY922 in combination with DOX in MCF-7 cell line. The possible mechanism of these cytotoxic effects might be related to apoptosis induction, cell growth inhibition and likewise downregulation of VEGF as the main angiogenic factor. It could be concluded that these altered levels of caspase 3 and VEGF can predict the treatment outcome in this model.

## References

[B1] Adams J, Carder P J, Downey S (2000). Vascular endothelial growth factor (VEGF) in breast cancer: comparison of plasma, serum, and tissue VEGF and microvessel density and effects of tamoxifen. Cancer Res.

[B2] Amiri B, Ebrahimi-Far M, Saffari Z (2016). Preparation, characterization and cytotoxicity of silibinin-containing nanoniosomes in T47D human breast carcinoma cells. Asian Pac J Cancer Prev.

[B3] Arshad Z, Rezapour-Firouzi S, Mohammadian M (2018). The sources of essential fatty acids for allergic and cancer patients; a connection with insight into mammalian target of rapamycin: A narrative review. Asian Pac J Cancer Prev.

[B4] Atari-Hajipirloo S, Nikanfar S, Heydari A (2017). Imatinib and its combination with 2,5-dimethyl-celecoxibinduces apoptosis of human HT-29 colorectal cancer cells. Res Pharm Sci.

[B5] Bradford MM (1976). A rapid and sensitive method for the quantitation of microgram quantities of protein utilizing the principle of protein-dye binding. Anal Biochem.

[B6] Canonici A, Qadir Z, Conlon N T (2018). The HSP90 inhibitor NVP-AUY922 inhibits growth of HER2 positive and trastuzumab-resistant breast cancer cells. Invest New Drugs.

[B7] Chun K-H, Park J H, Fan S (2017). Predicting and overcoming chemotherapeutic resistance in breast Cancer. Adv Exp Med Biol.

[B8] Ebrahimifar M, Roudsari MH, Kazemi SM (2017). Enhancing effects of curcumin on cytotoxicity of paclitaxel, methotrexate and vincristine in gastric cancer cells. Asian Pac J Cancer Prev.

[B9] Jensen M R, Schoepfer J, Radimerski T (2008). NVP-AUY922: a small molecule HSP90 inhibitor with potent antitumor activity in preclinical breast cancer models. Breast Cancer Res.

[B10] Jokar F, Mahabadi J A, Salimian M (2019). Differential expression of HSP90β in MDA-MB-231 and MCF-7 cell lines after treatment with doxorubicin. J Pharmacopunct.

[B11] Kanaani L, Javadi I, Ebrahimi Far M (2017). Effects of cisplatin-loaded niosomal nanoparticleson BT-20 human breast carcinoma cells. Asian Pac J Cancer Prev.

[B12] Maroufi N F, Vahedian V, Akbarzadeh M (2020). The apatinib inhibits breast cancer cell line MDA-MB-231 in vitro by inducing apoptosis, cell cycle arrest, and regulating nuclear factor-κB (NF-κB) and mitogen-activated protein kinase (MAPK) signaling pathways. Breast Cancer.

[B13] Mayor-López L, Tristante E, Carballo-Santana M (2014). Comparative study of 17-AAG and NVP-AUY922 in pancreatic and colorectal cancer cells: are there common determinants of sensitivity?. Clin Transl Oncol.

[B14] Miyata Y, Nakamoto H, Neckers L. (2013). The therapeutic target Hsp90 and cancer hallmarks. Curr Pharm Des.

[B15] Mohamadi N, Kazemi S M, Mohammadian M (2017). Toxicity of cisplatin-loaded poly butyl cyanoacrylate nanoparticles in a brain cancer cell line: Anionic polymerization results. Asian Pac J Cancer Prev.

[B16] Mohammadian M, Zeynali-Moghaddam S, Ansari M HK (2019). Dihydropyrimidine dehydrogenase levels in colorectal cancer cells treated with a combination of heat shock protein 90 inhibitor and oxaliplatin or capecitabine. Adv Pharm Bull.

[B17] Mohammadian M, Zeynali S, Azarbaijani AF (2017). Cytotoxic effects of the newly-developed chemotherapeutic agents 17-AAG in combination with oxaliplatin and capecitabine in colorectal cancer cell lines. Res Pharm Sci.

[B18] Moradi Z, Mohammadian M, Saberi H (2019). Anti-cancer effects of chemotherapeutic agent; 17-AAG, in combined with gold nanoparticles and irradiation in human colorectal cancer cells. Daru.

[B19] Niu G, Chen X (2010). Vascular endothelial growth factor as an anti-angiogenic target for cancer therapy. Curr Drug Targets.

[B20] Park H-K, Lee J-E, Lim J (2014). Combination treatment with doxorubicin and gamitrinib synergistically augments anticancer activity through enhanced activation of Bim. BMC Cancer.

[B21] Poy D, Ebrahimi Shahemabadi H, Akbarzadeh A (2018). Carboplatin liposomal nanoparticles: Preparation, characterization, and cytotoxicity effects on lung cancer in vitro environment. Int J Polym Mater.

[B22] Proia D A, Zhang C, Sequeira M (2014). Preclinical activity profile and therapeutic efficacy of the HSP90 inhibitor ganetespib in triple-negative breast cancer. Clin Cancer Res.

[B23] Smith L, Watson MB, O’Kane SL (2006). The analysis of doxorubicin resistance in human breast cancer cells using antibody microarrays. Mol Cancer Ther.

[B24] Sajjadiyan Z, Ghadernejad H, Milani AT (2016). Preparation of silibinin loaded pegylatedniosomal nanoparticles and investigation of its effect on MCF-10A human breast cancer cell lines. Der Pharmacia Lett.

[B25] Van Scheltinga AGT, Berghuis P, Nienhuis HH (2014). Visualising dual downregulation of insulin-like growth factor receptor-1 and vascular endothelial growth factor-A by heat shock protein 90 inhibition effect in triple negative breast cancer. Eur J Cancer.

[B26] Zeynali M, Kheradmand F, Mohammadian M (2018). A molecular basis for the synergy between 17-allylamino-17-demethoxy geldanamycin with Capecitabine and Irinotecan in human colorectal cancer cells through VEFG and MMP-9 gene expression. Gene.

